# Dynamic distribution of gallbladder microbiota in rabbit at different ages and health states

**DOI:** 10.1371/journal.pone.0211828

**Published:** 2019-02-04

**Authors:** Yawei Xing, Jianping Liu, Fanggen Lu, Li Wang, Ying Li, Chunhui Ouyang

**Affiliations:** Department of Gastroenterology, the Second Xiangya Hospital, Central South University, Changsha, Hunan, China; Kyungpook National University, REPUBLIC OF KOREA

## Abstract

The internal environment of the gallbladder has been considered extremely unfavorable for bacterial growth, and the microbial profile of the gallbladder still unknown. By high-throughput sequencing of the bacterial 16S rRNA gene, we studied the microbial profile of the gallbladder from healthy rabbits before and after weaning. Moreover, we investigated the difference of microbiota between the gallbladder and gut. Our results showed that the gallbladder was dominantly populated by *Firmicutes*, *Bacteroidetes*, *Proteobacteria*, and *Actinobacteria* in the phylum throughout the developmental stages of rabbits. The adult rabbits showed higher species richness and exhibited higher bacterial diversity than rabbits before weaning based on the results of alpha diversity. Beta diversity analyses indicated differences in the bacterial community composition between different developmental stages. In the comparison of the gallbladder and feces, *Firmicutes* and *Bacteroidetes* were dominant in the phylum, as they were present in about 61% and 21% of the feces, respectively. Conversely, in the gallbladder, *Firmicutes* was the most dominant (about 41%), and *Bacteroidetes* and *Proteobacteria* were present in about 16% and 22% of the gallbladder, respectively. The Unweighted UniFrac Principal Coordinate Analysis results illustrated samples clustered into 2 categories: the gallbladder and feces. Our study might provide a foundation for knowledge on gallbladder microbiota for the first time and a basis for further studies on gallbladder and intestinal health.

## Introduction

The gastrointestinal tract (GIT) of humans and animals colonizes enormous microbiota that forms a dynamic ecosystem. This complex microbiota forms a barrier against exogenous harmful substances and provides metabolic, immunological, and neurological benefits to the host [1–4]. To date, most studies about bacterial diversity and function have focused on the colon because of easy availability of samples (through feces or biopsies) and the high abundance of bacteria in the colon. In contrast, other parts of the GIT (e.g., the stomach, gallbladder, and small intestine) are difficult to access to obtain biological specimens because of technical problems, making us less aware of the characteristics of microbiota in these areas. However, thanks to the advancement of new technologies, an unexpected and wide-ranging microbial population has been revealed in this part of the GIT. Studies have indicated that GIT microbiota is a reflection of evolutionary selection pressures at the level of the host and microbial cells. The GIT microbiota is closely involved in many aspects of normal host physiology from the nutritional status to behavioral and stress responses [5].

Previously, the stomach was considered as a sterile organ because of the barrier effect of hydrochloric acid on the inlet bacteria. The discovery of *Helicobacter pylori* led researchers to reconsider the potentiality of the stomach as a bacterial niche [6, 7]. With the invention of next generation sequencing technology, each bacterial phylum, class, and genus have been found in healthy stomachs [8, 9].

Traditionally, like the stomach, the gallbladder is an auxiliary digestive organ, and its internal environment has been considered extremely unfavorable for bacterial growth. The most important material in the gallbladder is bile, which acts as a biological detergent for emulsifying and solubilizing lipids, and it is essential for fat digestion. At the same time, the role of bile detergents gives it effective antimicrobial properties [10]. Numerous studies have shown that bile acid, the major constituent of bile, can have a role in antimicrobial activity through the following ways. Bile acids (BAs) have antimicrobial properties in dissolving the bacterial membrane and damaging bacterial DNA [10, 11]. The administration of bile acid in the diet of rat can induce phylum-level alterations of the gut microbiota [12]. Obstruction of bile acid flow results in bacterial proliferation in the small intestine and mucosal injury, indicating that bile acid plays a key role in preventing bacterial proliferation [13–15].

Although the gallbladder environment is unsuitable for bacterial growth, recent evidence suggests that certain bacteria can colonize in the gallbladder and are closely related to the pathogenesis of gallstones and cholecystitis, for example, *Salmonella spp*. and *Listeria monocytogenes* [11, 16, 17]. In addition, with the development of technology, many bacteria in gallstones of patients with a non-inflammatory status have been found [18–21]. Therefore, at least some bacteria are able to colonize in the extreme environment of bile [22–24]. However, the global bacterial diversity of the gallbladder of healthy hosts has not been fully understood so far.

Rabbits’ unique digestive properties and microbial communities, which can help them to adapt to high-fiber foods while also making them susceptible to various diseases have been demonstrated. The objectives of this study were to describe the microbiome of gallbladder samples collected from healthy rabbits, and to systematically study the transition of gallbladder microbiota from infant to adult rabbits in order to evaluate the shift of microbes during maturation and investigate the difference between the gallbladder and gut.

## Materials and methods

### Animal model and sample collection

Healthy, male New Zealand White rabbits (Medical Experimental Animal Center of Hunan Province, China) (n = 12) were used in this study. Rabbits were weaned at 4 weeks of age. Five of them were young rabbits (3 weeks old) before weaning. The other adult rabbits (n = 7, 18 weeks old) were fed regular rabbit chow. All rabbits were euthanized by barbiturate overdose (intravenous injection, 150 mg/kg pentobarbital sodium) for tissue collection. Twelve gallbladders were resected from them on a super clean bench. Once the abdominal cavity of the animal was opened, the gallbladder was ligated from the common bile duct, it was removed using a sterile scalpel, and it was placed into a separate sterile container. Samples were stored at -80°C until use. Fecal samples from adult rabbits were collect in a fasting condition and stored at -80°C until use.

Methods used in this experiment were in line with the guidelines for the care and use of experimental animals developed by the Chinese Society of Laboratory Animal Science, and this study was approved by the Animal Research Committee, Central South University, Hunan, China.

### DNA extraction

Microbial genomic DNA was extracted from 1000 mg of each gallbladder sample and 500 mg of each fecal sample. Microbial genomic DNA was extracted from the samples using the QiaAmp DNA Stool Mini Kit (Qiagen, Hilden, Germany) following the manufacturer’s instructions. The quality of DNA was detected by 0.8% agarose gel electrophoresis. The concentration and purity of DNA were examined using spectrophotometry on NanoDrop (Fisher Scientific, Schwerte, Germany).

### Gallbladder microbes’ 16S rRNA sequencing

The V4 hypervariable region of 16S rRNA was amplified by polymerase chain reaction (PCR) using specific barcoded primers (515F: 5’-GTGCCAGCMGCCGCGGTAA-3’ and 806R: 5’-GGACTACHVGGGTWTCTA AT-3’) [25]. All PCR procedures were performed using the Phusion High-Fidelity PCR Master Mix (New England Biolabs, Ipswich, MA, USA) with the PCR programs. The PCR was run in a total reaction volume of 25 μL. Each reaction mixture contained 2.5-μL 10×PCR buffer, 2-μL dNTP, 2-μL forward and reverse primers (1 μL each), 0.5-μL Taq DNA polymerase, 2-μL DNA template, and 11-μL sterile water. The PCR conditions were as follows: initial denaturation at 94°C for 5 minutes, followed by 25 cycles of 94°C denaturation for 30 seconds, 50°C of annealing for 30 seconds, 72°C of extension for 30 seconds, and a final extension at 72°C for 7 minutes [26]. A mixture of PCR products was purified using the Qiagen Gel Extraction Kit (Qiagen, Hilden, Germany). Then the PCR products were used to generate a sequencing library with the TruSeq DNA PCRFREE Sample Preparation Kit (Illumina HiSeq 2500 platform, San Diego, CA, USA). Finally, the library was sequenced on the Illumina HiSeq 2500 platform and a 250-bp paired end read was generated.

### Taxonomy classification

Similar to our previous methods, 16S raw data for all samples were processed and analyzed using QIIME pipeline (1.9.1) [25, 27]. Raw data obtained by sequencing contained a certain proportion of disturbance data (dirty data). In order to make the results of information analysis more accurate and reliable, the raw data were first spliced and filtered to obtain valid data (clean data). Then the clean data were subjected to chimera filtration to obtain effective data for subsequent analysis. We clustered effective tags for all samples and clustered sequences into operational taxonomic units (OTUs) with a 97% sequence identity. A representative sequence for each OTU was selected and classified using the Greengene database gg_13_8. According to the analysis of OTUs, the species with the highest abundance at each taxonomic level (phylum, class, order, family, and genus) for each sample was selected to generate the relative abundance of species. The bacterial abundance and diversity of the samples were determined by calculating the Shannon-Weaver diversity index, ACE, and Chao 1 after taking into account the number and uniformity of the bacterial species. Sequence alignment and construction of the phylogenetic tree was performed using the PyNAST method [28]. Then the phylogenetic tree was used for Unweighted UniFrac Principal Coordinate Analysis (PCoA). This study used PCoA to analyze the similarity of the composition of gallbladder flora in pre-weaning young rabbits and adult rabbits. According to results of the OTU clustering analysis and research needs, the common and unique OTUs among different samples (groups) were analyzed. When the number of samples (groups) was less than 5, the Venn Graph was drawn. The microbial function was predicted by FAPROTAX [29].

### Statistical analysis

The measurement data obtained in the study are expressed as mean±standard deviation, and the component difference was analyzed by one-way analysis of variance (ANOVA). Statistical analysis was performed using SPSS 22.0 software (IBM Corp., Armonk, NY, USA). The difference was considered statistically significant when P<0.05.

The DOI for our protocol is dx.doi.org/10.17504/protocols.io.xarfid6.

## Results

### Sample and baseline data

**Fig 1:**
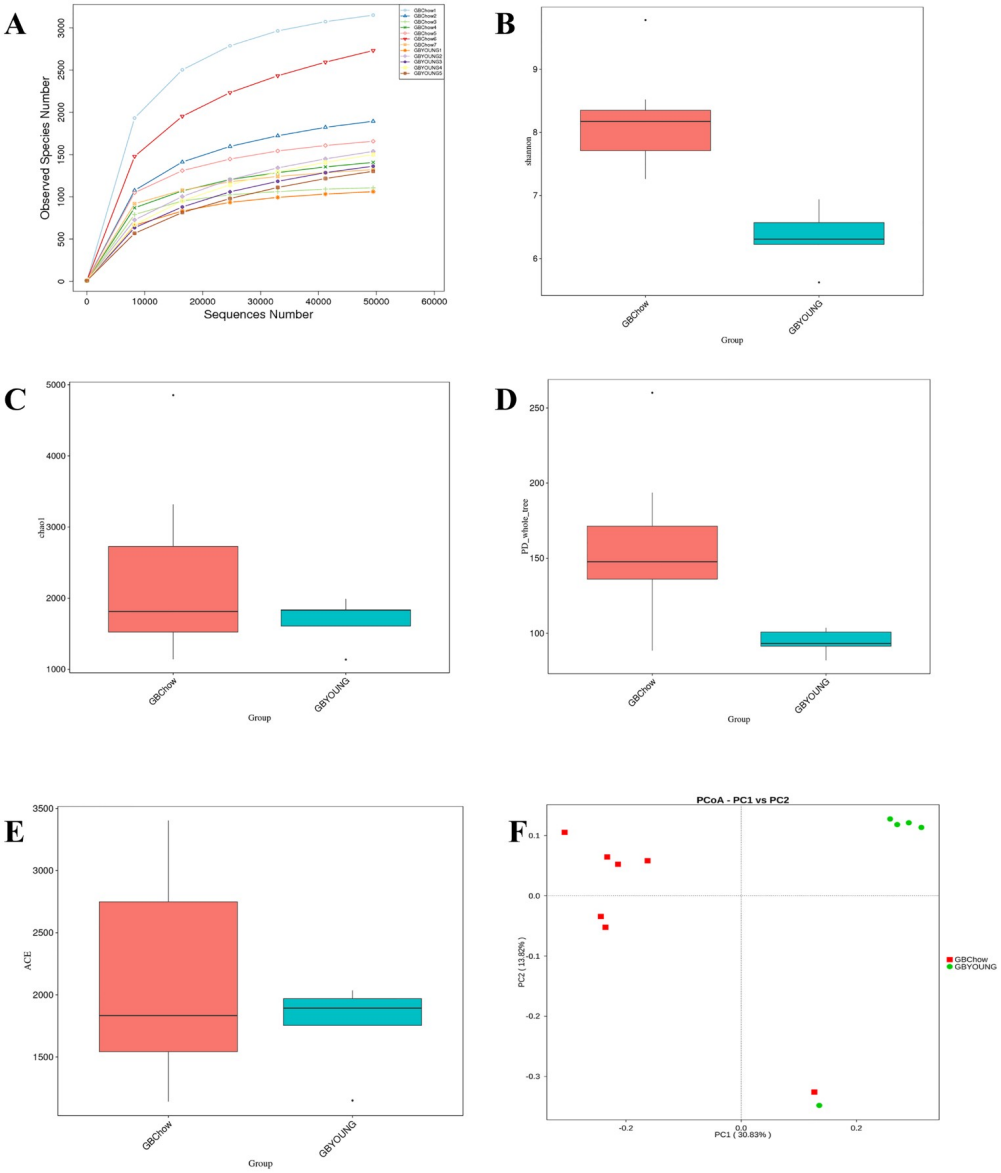
Measures of microbial diversity of gallbladder samples of the 2 age groups. A: Rarefaction curves (at 97% similarity level) of each gallbladder sample. B: Box plot of the Shannon-Weaver index. C: Box plot of the Chao-1 index. D: Box plot of phylogenetic diversity whole tree. E: Box plot of the ACE index. F: Unweighted UniFrac Principal Coordinate Analysis. GBChow: adult healthy rabbits; GBYOUNG: healthy rabbits before weaning.

We collected 12 samples from the gallbladder (5 from young rabbits and 7 from adult rabbits) and 7 samples from fecal matter (all from adult rabbits). As a rabbit’s gallbladder is too small to distinguish between gallbladder mucus and bile, we resected the whole gallbladder as one sample. For each sample, the V4 hypervariable region of the bacterial 16S rRNA gene sequence was amplified using PCR. After filtering out the sequences of low mass and short length, 1,404,179 high-quality reads were obtained (376,298 of young rabbits’ gallbladder, 420,770 of adult rabbits’ gallbladder, and 607,111 of adult rabbits’ feces). In this series of data, we identified 34,307 OTUs based on the conventional criterion of a 97% sequence identity (equal to species level), with samples harboring an average of 1,597 OTUs. The good coverage index (sequencing depth index) for all samples ranged from 0.984 to 0.997. Analysis based on the results of rarefaction curves and good coverage indices demonstrated that the sequencing depth in this study was sufficient to reflect the bacterial composition of the different sets of samples. The raw data of the samples are shown in S1 Table, and the rarefaction curve of each sample is presented in Fig 1A.

### Changes in gallbladder microbiota along with body development

Alpha diversity reflected the community richness and diversity of gallbladder microbiota. The Shannon-Wiener indices of young and adult rabbits were 6.33±0.48 and 8.19±0.81, respectively. Compared with young rabbits, the Shannon index of adult rabbits was significantly increased (P<0.05) (Fig 1B). The Shannon-Wiener index directly reflects the heterogeneity of a community based on the number of species present and relative abundance. The adult rabbits showed greater diversity than the young rabbits. The Chao-1 and ACE indices were also increased in adult rabbits, but this was not statistically significant (P>0.05) (Fig 1C and 1E). The specific values of alpha diversity are shown in Table 1 and S2 Table. Moreover, good coverage values were 99.2% and 99.3% for young and adult rabbits, respectively, indicating excellent coverage (Table 1). These results indicate that the richness and evenness of microbes in the gallbladder varied during the developmental stage of rabbits.

**Table 1:** Alpha diversity of the gallbladder in rabbits before and after weaning.

	Young Rabbits GB (n = 5)	Adult Rabbits GB (n = 7)
Shannon*	6.33±0.48	8.19±0.81
Observed species*	1353±189.3	1896±765.9
**Chao 1**	1680.9±333	2330.1±1315
**ACE**	1760.6±319.7	2317.3±830.1
PD whole tree*	94.2±8.52	158.7±54.3
**Good coverage (%)**	99.2±0.3	99.3±0.5

* Significant difference of P<0.05, based on oneway analysis of variance by ranks. Young Rabbist: rabbits before weaning; adult rabbits: rabbits after weaning; PD: phylogenetic diversity.

The PCoA clearly showed differences between all sampled rabbits individually and by group. Our results illustrated differences in the distribution of the microbial population at different ages. Before weaning, young rabbits were significantly different from adult rabbits (Fig 1F).

**Fig 2:**
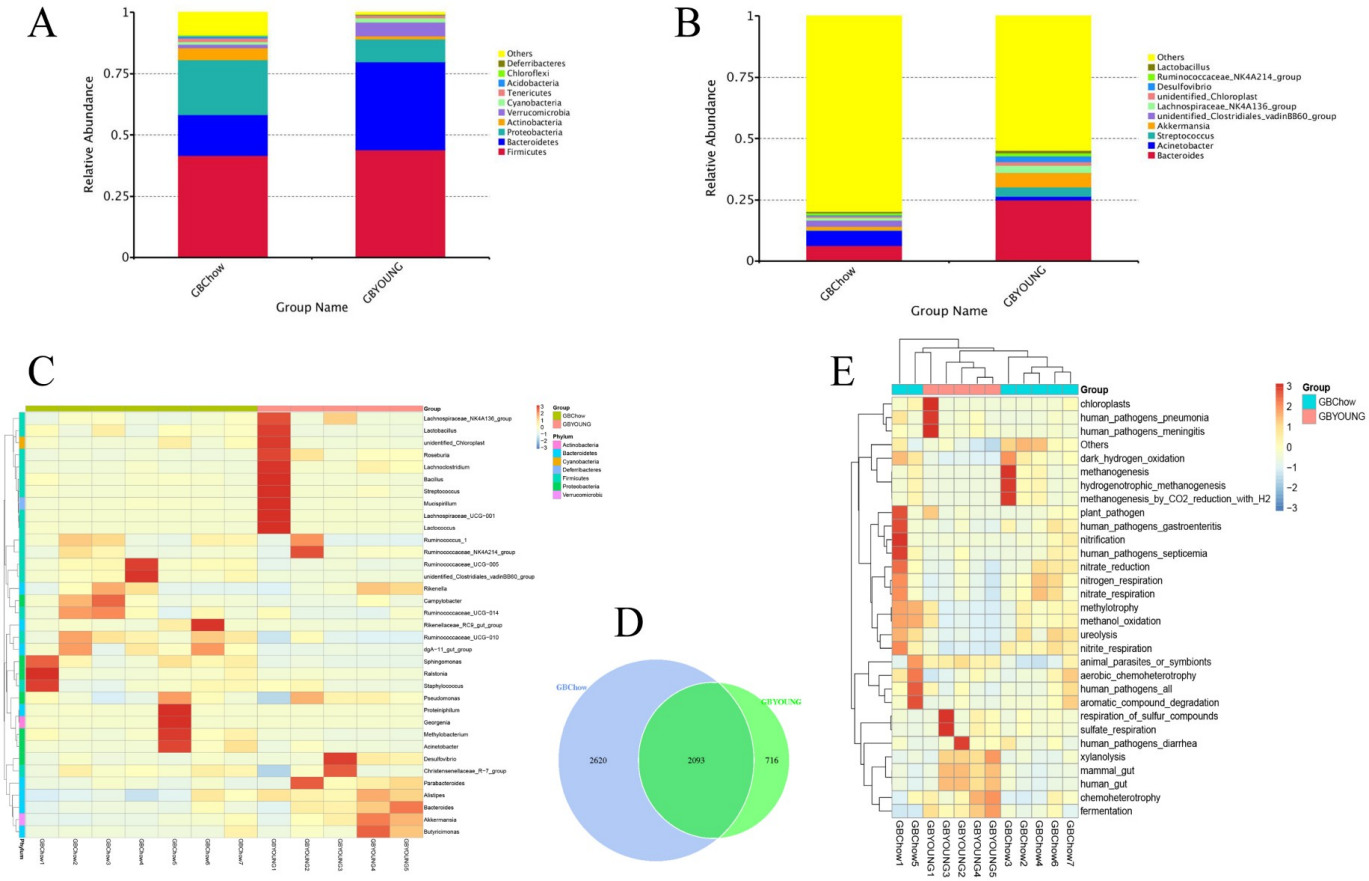
Profiles of gallbladder microbes in rabbits of the 2 age groups. A: Phyla level comparisons of the microbial components. B: Genera level comparisons of microbial components. C: Heat map of hierarchy cluster results for the abundance of genus. D: Venn diagram described the number of features that are distinct and shared across the young and adult rabbits. E: Functional analysis of the microbes used by FAPROTAX. GBChow: adult healthy rabbits; GBYOUNG: healthy rabbits before weaning.

Throughout the developmental stage of rabbits, *Firmicutes*, *Bacteroidetes*, *Proteobacteria*, and *Actinobacteria* were the four dominant phyla of gallbladder microbiota, with the most dominant being *Firmicutes*. The relative abundances of *Firmicutes* and *Bacteroidetes* were higher in young rabbits than in adult rabbits (44% versus [vs.] 42%, P>0.05 and 36% vs. 16%, P<0.05). Conversely, the relative abundances of *Proteobacteria* and *Actinobacteria* in young rabbits were inferior to those in adult rabbits (10% vs. 22%, P<0.05 and 1% vs. 5%). Fig 2A shows the relative abundance of each of these phyla within the animals.

Further, the cluster analysis, based on the relative abundance of the genera, showed differences between the dominant species of pre-weaning young and adult rabbits (Fig 2B). At the genus level, the bacterial genera were significantly different between young and adult rabbits. The dominant genera in young rabbits included *Bacteroides*, *Streptococcus*, *Akkermansia*, *Lachnospiraceae* (24.9%, 3.9%, 5.8%, and 2.9%, respectively). However, in the adult rabbits, *Bacteroides*, *Acinetobacter*, and *Clostridiales* were relatively abundant (6.3%, 6.2%, and 2.5%, respectively) (Fig 2B and S3 Table).

The study further revealed that 12 samples could be divided into 3 different clusters based on the heat map. The microbiota of young rabbits was predominant in both ends of the cluster, and the microbiota of adult rabbits was predominant in the middle cluster (Fig 2C). The shared taxa among all rabbits within groups were considered as core OTUs. The number of core OTUs shared by all rabbits within the 2 groups was 2,093. We found that the core OTUs account for about 38.6% of all the bacterial OTUs. Moreover, 2,620 OTUs were found uniquely in the adult rabbits, and 716 unique OTUs in the young rabbits (Fig 2D).

To investigate the differences in microbial function between the gallbladder of young and adult rabbits, we performed functional analysis of the microbes using FAPROTAX. Compared with young rabbits, adult rabbits’ gallbladder microorganisms had a higher functional richness, involving nitrification, nitrate reduction, nitrite respiration, methanol oxidation, and ureolysis. Compared with adult rabbits, young rabbits had microbes and pathogenic bacteria associated with a lower immune system (Fig 2E).

### Microbiota profile in the gallbladder and gut of adult rabbits

**Fig 3:**
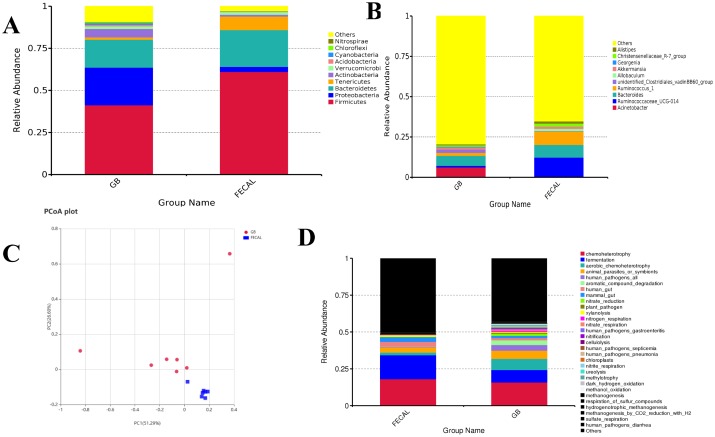
Microbial profiles of the gallbladder and feces. A: Phyla level comparisons of the microbial components. B: Genera level comparisons of microbial components. C: Unweighted UniFrac Principal Coordinate Analysis. D: Functional analysis of the microbes used by FAPROTAX. GB: gallbladder; FECAL: feces.

The microbial structure became stable in the host at maturity, so we investigated the microbiota profile in the gallbladder and feces of adult rabbits. *Firmicutes* and *Bacteroidetes* were dominant phyla, as they were present in about 61% and 21% of the feces, respectively. Conversely, in the gallbladder, *Firmicutes* was the most dominant (about 42%), and *Bacteroidetes* and *Proteobacteria* were present in about 16% and 22% of the gallbladder, respectively (Fig 3A). At the genus level, the bacterial genera were significantly different between the gallbladder and feces. The dominant genera in the gallbladder included *Bacteroides*, *Acinetobacter*, and *Clostridiales* (6.3%, 6.2%, and 2.5%, respectively). However, in feces, *Ruminococcaceae*, *Bacteroides*, and *Ruminococcus* were relatively abundant (12%, 7.9%, and 8.2%, respectively) (Fig 3B and S4 Table). The abundance of genera was mostly higher in the feces than in the gallbladder. For example, *Ruminococcaceae* was 100 times more abundant in feces than in the gallbladder. Although some genera had a lower abundance in feces, they were barely detected in the gallbladder, such as *Allobaculum*. The PCoA results illustrated samples clustered into 2 categories: the gallbladder and gut (Fig 3C). In order to investigate the difference of microbiota function between the gallbladder and gut, we performed functional analysis by FAPROTAX (Fig 3D). Compared with the gut, microbiota of the gallbladder had a higher abundance of functions involved in the metabolic pathways such as chemoheterotrophy, degradation, and respiration. Conversely, the gut contained much more microbiota associated with fermentation. Moreover, the gallbladder seemed to contain higher immune microbes.

## Discussion

The results of the study, which show the opposite of the traditional view of the gallbladder environment in humans, demonstrates for the first time the presence of abundant microbes in the gallbladder of healthy rabbits and changes in gallbladder microbiota along with body development. Moreover, we compared the composition of the profile of microbiota of the gallbladder and gut.

High concentrations of BAs in the gallbladder produce antibacterial effects through antimicrobial membrane properties and DNA damage. Therefore, the gallbladder has been considered sterile under non-pathogenic conditions for a long time [11]. As we know, to date, research has covered many aspects of mammalian microbiota, including the stomach, small intestine, and colon. However, no study on the profile of the gallbladder microbiota in healthy rabbits has been published. This is the first study to use a culture-independent 16S rRNA high-throughput sequencing to explore the composition of a healthy rabbit gallbladder. Through OTU clustering, ANOVA, heat map, and Unweighted UniFrac PCoA analysis, we identified differences in gallbladder microbes and internal composition diversity in healthy rabbits of different ages, providing unprecedented insight into the gallbladder microbiota. By applying more advanced molecular methods in our experiments, 797,068 high-quality reads of the gallbladder were obtained, and then we identified 22,566 OTUs based on the conventional criterion of a 97% sequence identity. Compared with previous studies on the gut microbiota of New Zealand white rabbits [30], the bacterial diversity seemed to be higher in the gallbladder than in the small intestine, surprisingly.

Enterohepatic circulation is a normal physiological phenomenon of the digestive tract. About 5% of BAs can escape from the enterohepatic circulation, flow into the colon, and exert strong selective pressure on colonic bacteria to shape the profile of gut microbiota [10, 12, 31, 32]. High-fat diets can change the bile composition through hepatic BA, which seems to be suitable to the growth of inflammatory intestinal microorganisms. As the dominant component of the gallbladder, BA is thought to be composed of amphiphilic molecules with strong antibacterial activity. Therefore, it was considered in the past that the gallbladder is sterile under non-pathogenic conditions. Only recently, evidence has shown that various enteric bacteria have formed a defense mechanism that confers BA resistance [11, 16]. Microbiota has been detected in the bile of patients with acute cholecystitis. Furthermore, several studies using electron microscopy, culture, and testing of bacterial DNA demonstrated that microbiota, including Gram-negative and Gram-positive bacteria, are present in non-inflammatory gallstones [22–24].

Notably, it has been reported that when the pH of bile is similar to the natural pH (pH = 7) of the gallbladder, the bile does not serve as a harsh environment for bacterial growth of broad-spectrum Gram-negative and Gram-positive bacteria; thus, a specific molecular detoxification system is not needed [33]. However, after the bile is discharged into the duodenum and mixed with chyme from the stomach, its local pH decreases to 5.2. In this case, the bile becomes toxic, and the bacteria need special proteases to survive, such as *L*. *monocytogenes* [33]. A recent study showed that bacteria normally present in the intestine may invade the biliary tract through the bloodstream of the hepatic portal vein or by rising from the duodenum [34]. Costello et al. [35] reported that the niches in the human microbiome are comprised of an interconnected community network that undergoes constant exchanges, not an isolated environment. In addition to specific bile resistance mechanisms (including the bile efflux system and bile saline hydrolase), some bacteria also show potential to assimilate and metabolize certain components of bile. Our study findings [33] confirm this phenomenon. We found that during the growth of rabbits, *Firmicutes*, *Bacteroidetes*, *Proteobacteria*, and *Actinobacteria* are always the four dominant phyla of gallbladder microbiota, which is consistent with the structure of the gut microbiota of rabbits in early research studies.

It is thought that the flexible distribution of gut microbiota is affected by host factors during development, such as breast-feeding, sex, and genotype [36–40]. However, there is no research on this aspect of the gallbladder microbiota until now. In rabbits, our study results demonstrated that the composition of gallbladder microbes changed during rabbit development. Microbial profiles were significantly different between pre-weaning young rabbits and adult rabbits. According to reports, the ratio of the *Firmicutes/Bacteroidetes* of mammal’s gut microbiota varies with age [26, 41], and the same trend was observed in our study of rabbit gallbladder microbiota. The increase of the *Firmicutes/Bacteroidetes* ratio with rabbit’s growth, which we observed herein, was mainly due to the reduction of *Bacteroidetes* in adult rabbits. Contrary to the sudden decrease in the number of *Bacteroidetes*, the number of *Proteobacteria* in the gallbladder of rabbits increased significantly with age. In our study, the diversity and abundance of gallbladder microbiota also increased with age. The changing diversity index of Shannon and abundance indexes of Chao 1 and ACE and the PD whole tree demonstrated the evenness and richness in mature adult rabbits compared to pre-weaning rabbits. This was consistent with the phenomenon that the gut microbiota of mammals significantly changes before and after weaning [26, 42–44]. During the weaning transition period, the diet of a young rabbit changes from highly digestible milk to solid feed. Therefore, in addition to age factors, weaning stress and changes in food composition might also contribute to the significant changes in gallbladder microbes.

No study has compared the gut microflora profile and gallbladder microbial profile. As the closest structure to feces, the colon completes the final chyme processing. From our viewpoint, feces share most of the microbes with the colon [45]. Thus, we investigated the microbiota profile of the gallbladder and feces in adult rabbits. In our study, we identified 14,736 OTUs of the gallbladder and 11,741 OTUs of the feces in rabbits after weaning based on the conventional criterion of a 97% sequence identity. Distinct microbial communities between the gut and gallbladder were found by PCoA. *Firmicutes* and *Bacteroidetes* were dominant phyla, as they were present in about 61% and 21% of feces, respectively. Conversely, in the gallbladder, *Firmicutes* was the most dominant (about 42%), and *Bacteroidetes* and *Proteobacteria* were present in about 16% and 22% of the gallbladder, respectively. At the genera level, the dominant genera in the gallbladder included *Bacteroides*, *Acinetobacter*, and *Clostridiales* (6.3%, 6.2%, and 2.5%, respectively). However, in feces, *Ruminococcaceae*, *Bacteroides*, and *Ruminococcus* were relatively abundant (12%, 7.9%, and 8.2%, respectively). We speculated that owing to the different functions of the gallbladder and intestinal tract, the colonization of bacteria was significantly different from the phylum to genus. The colon is mainly responsible for microbial fermentation, whereas the gallbladder, as organ for storing bile, is associated with metabolism [46]. In our research, the results of microbial function prediction by FAPROTAX showed microbiota of gallbladder own high abundance of functions involed in metabolic pathways compared with gut. Thus, our results signified, for the first time, that host distribution of microbes was segmented at the demarcation point of the gallbladder and gut. The gallbladder is a blind organ with only one outlet that connects the duodenal papilla to the gut, which leads us to guess that its microbiota come from the small intestine. Further research is needed to determine the different profiles of the gallbladder and small intestine.

To the best of our knowledge, this is the first study to demonstrate that a complex microbiota is colonized in the gallbladder of healthy New Zealand white rabbits. Additionally, we found that the profile of microbiota changes with age development. This unexpected fact raises the hypothesis that the microbial populations in the gallbladder may be affected by factors that are currently unknown, and this dysbiosis process may be associated with gallbladder lesions. Because of the adjacent anatomy between the gallbladder and gut, further studies of gallbladder microbiota in humans and its effect on gallbladder and intestinal health are necessary.

## Supporting information

S1 Table Raw data summary. (DOCX)

S2 Table Alpha diversity. Specific alpha diversity values of the gallbladder samples.
(DOCX)

S3 Table Bacterial genera of the gallbladder. The relative abundance of bacterial genera of the gallbladder in rabbits before and after weaning at the genus level.
(DOCX)

S4 Table Bacterial genera of the gallbladder and feces. The relative abundance of bacterial genera of the gallbladder and feces in rabbits after weaning at genus level.
(DOCX)
