# Supplementary material for: Dynamic distribution of gallbladder microbiota in rabbit at different ages and health states
Source: PLoS One. 2019 Feb 4;14(2):e0211828. doi: 10.1371/journal.pone.0211828 (PMC6361460; doi:10.1371/journal.pone.0211828)
Supplement: S1 Table — (DOCX) [file pone.0211828.s001.docx]

| Samples | Effective Tags | Avglen (nt) | OTUs | Coverage (%) |
| --- | --- | --- | --- | --- |
| GBYOUNG1 | 57262 | 253 | 1152 | 0.997 |
| GBYOUNG2 | 76867 | 252 | 1187 | 0.991 |
| GBYOUNG3 | 77186 | 249 | 1682 | 0.992 |
| GBYOUNG4 | 80338 | 256 | 1836 | 0.99 |
| GBYOUNG5 | 84645 | 253 | 1643 | 0.99 |
| GBCHOW1 | 61424 | 252 | 3381 | 0.992 |
| GBCHOW2 | 64509 | 251 | 2167 | 0.992 |
| GBCHOW3 | 72634 | 253 | 1152 | 0.998 |
| GBCHOW4 | 55139 | 252 | 1600 | 0.995 |
| GBCHOW5 | 53427 | 254 | 1877 | 0.995 |
| GBCHOW6 | 54097 | 251 | 2732 | 0.984 |
| GBCHOW7 | 59540 | 253 | 1467 | 0.996 |
| FECESCHOW1 | 92414 | 252 | 1425 | 0.99 |
| FECESCHOW2 | 80314 | 253 | 1410 | 0.991 |
| FECESCHOW3 | 82099 | 251 | 3114 | 0.992 |
| FECESCHOW4 | 94482 | 253 | 1467 | 0.997 |
| FECESCHOW5 | 80360 | 252 | 1397 | 0.993 |
| FECESCHOW6 | 85748 | 254 | 1431 | 0.994 |
| FECESCHOW7 | 91694 | 253 | 1497 | 0.996 |

**Supplementary table 1.Raw Data summary**
