# Supplementary material for: Dynamic distribution of gallbladder microbiota in rabbit at different ages and health states
Source: PLoS One. 2019 Feb 4;14(2):e0211828. doi: 10.1371/journal.pone.0211828 (PMC6361460; doi:10.1371/journal.pone.0211828)
Supplement: S2 Table — Specific alpha diversity values of the gallbladder samples. (DOCX) [file pone.0211828.s002.docx]

| Samples | ACE | Chao1 | Shannon | Observed  species | PD whole tree | Coverage (%) |
| --- | --- | --- | --- | --- | --- | --- |
| GBYOUNG1 | 1149.211 | 1137.007 | 6.939 | 1062 | 81.97 | 99.7 |
| GBYOUNG2 | 1969.741 | 1832.944 | 6.57 | 1538 | 100.776 | 99.1 |
| GBYOUNG3 | 1754.624 | 1608.676 | 6.308 | 1362 | 93.17 | 99.2 |
| GBYOUNG4 | 2036.092 | 1989.493 | 6.225 | 1501 | 103.601 | 99 |
| GBYOUNG5 | 1893.213 | 1836.281 | 5.623 | 1303 | 91.394 | 99 |
| GBCHOW1 | 3325.292 | 3318.402 | 9.778 | 3151 | 260.14 | 99.2 |
| GBCHOW2 | 2172.346 | 2136.438 | 8.173 | 1895 | 147.622 | 99.2 |
| GBCHOW3 | 1140.746 | 1141.515 | 7.262 | 1107 | 88.416 | 99.8 |
| GBCHOW4 | 1623.404 | 1600.143 | 7.719 | 1408 | 148.97 | 99.5 |
| GBCHOW5 | 1833.49 | 1812.921 | 7.701 | 1658 | 136.284 | 99.5 |
| GBCHOW6 | 3404.358 | 4853.811 | 8.52 | 2732 | 193.634 | 98.4 |
| GBCHOW7 | 1461.361 | 1447.238 | 8.179 | 1323 | 135.782 | 99.6 |

**Supplemental Table 2.** Specific alpha diversity values of gallbladder samples
