# Supplementary material for: Dynamic distribution of gallbladder microbiota in rabbit at different ages and health states
Source: PLoS One. 2019 Feb 4;14(2):e0211828. doi: 10.1371/journal.pone.0211828 (PMC6361460; doi:10.1371/journal.pone.0211828)
Supplement: S3 Table — The relative abundance of bacterial genera of the gallbladder in rabbits before and after weaning at the genus level. (DOCX) [file pone.0211828.s003.docx]

| Taxonomy | Bacteroides | Acinetobacter | Streptococcus | Akkermansia | unidentified_Clostridiales_vadinBB60_group | Lachnospiraceae_NK4A136_group | unidentified_Chloroplast | Desulfovibrio | Ruminococcaceae_NK4A214_group | Lactobacillus |
| --- | --- | --- | --- | --- | --- | --- | --- | --- | --- | --- |
| GBYOUNG1 | 0.00469 | 0.00499 | 0.17554 | 0.00266 | 0.00034 | 0.07641 | 0.07466 | 0.00481 | 0.00061 | 0.03951 |
| GBYOUNG2 | 0.22477 | 0.03648 | 0.00319 | 0.03425 | 0.000363982 | 0.00754 | 0.00105 | 0.00603 | 0.04127 | 0.00222 |
| GBYOUNG3 | 0.19741 | 0.009706186 | 0.00243 | 0.05039 | 0.00028 | 0.04414 | 0.00016 | 0.06564 | 0.00499 | 0.00194 |
| GBYOUNG4 | 0.33324 | 0.01124 | 0.01175 | 0.11431 | 0.00016 | 0.00841 | 0.00131 | 0.02352 | 0.00772 | 0.00267 |
| GBYOUNG5 | 0.48674 | 0.00797 | 0.00204 | 0.08928 | 0.00024 | 0.00785 | 0.00012 | 0.01897 | 0.00764 | 0.00176 |
| GBCHOW1 | 0.01452 | 0.02127 | 0.00835 | 0.00330 | 0.00675 | 0.00418 | 0.00590 | 0.00253 | 0.00319 | 0.00876 |
| GBCHOW2 | 0.05035 | 0.01632 | 0.00125 | 0.00168 | 0.011501 | 0.01399 | 0.00091 | 0.00944 | 0.02081 | 0.00216 |
| GBCHOW3 | 0.05860 | 0.01747 | 6.07E-0 | 0.00896 | 0.01868 | 0.01616 | 0.00142 | 0.00374 | 0.01262 | 0.01383 |
| GBCHOW4 | 0.06111 | 0.01420 | 0.00220 | 0.00710 | 0.10426 | 0.00568 | 0.00218 | 0.00558 | 0.00269 | 0.00097 |
| GBCHOW5 | 0.03644 | 0.23089 | 0.00366 | 0.01666 | 0.00605 | 0.00400 | 0.02232 | 0.00235 | 0.00295 | 0.00811 |
| GBCHOW6 | 0.15886 | 0.03502 | 0.00376 | 0.02145 | 0.00849 | 0.01102 | 0.00218 | 0.00882 | 0.00586 | 0.00552 |
| GBCHOW7 | 0.05884 | 0.10076 | 0.00068 | 0.03551 | 0.02075 | 0.01650 | 0.01233 | 0.00590 | 0.00635 | 0.00663 |

**Supplementary Table S3.** The relative abundance of gallbladder’s bacterial genera in rabbits before and after weaning at genus level.
