# Supplementary material for: Dynamic distribution of gallbladder microbiota in rabbit at different ages and health states
Source: PLoS One. 2019 Feb 4;14(2):e0211828. doi: 10.1371/journal.pone.0211828 (PMC6361460; doi:10.1371/journal.pone.0211828)
Supplement: S4 Table — The relative abundance of bacterial genera of the gallbladder and feces in rabbits after weaning at genus level. (DOCX) [file pone.0211828.s004.docx]

**Supplementary Table S4.** The relative abundance of bacterial genera of gallbladder and feces in rabbits after weaning at genus level.

| Taxonomy | Acinetobacter | Ruminococcaceae_UCG-014 | Bacteroides | Ruminococcus_1 | unidentified_Clostridiales_vadinBB60_group | Allobaculum | Akkermansia | Georgenia | Christensenellaceae_R-7_group | Alistipes |
| --- | --- | --- | --- | --- | --- | --- | --- | --- | --- | --- |
| GBChow1 | 0.021092221 | 0.005971894 | 0.014230896 | 0.01273157 | 0.005311174 | 0.000787782 | 0.003176539 | 0.000559071 | 0.006276842 | 0.003379838 |
| GBChow2 | 0.01613682 | 0.017966507 | 0.049884374 | 0.030037356 | 0.007928642 | 0.000152474 | 0.001626388 | 0.000177886 | 0.010673172 | 0.004802927 |
| GBChow3 | 0.017432848 | 0.02030444 | 0.058702447 | 0.028004371 | 0.01481538 | 0.000508246 | 0.009021372 | 0 | 0.006962974 | 0.005641534 |
| GBChow4 | 0.014154659 | 0.008767249 | 0.060938731 | 0.00922467 | 0.094711697 | 0 | 0.006988387 | 0.000152474 | 0.008589362 | 0.00142309 |
| GBChow5 | 0.230260984 | 0.00307489 | 0.036415847 | 0.007954055 | 0.004574217 | 0.001473914 | 0.016645066 | 0.0290971 | 0.007471221 | 0.004599629 |
| GBChow6 | 0.034408274 | 0.00932632 | 0.156057025 | 0.01987243 | 0.005031638 | 0.000457422 | 0.021168458 | 0.000381185 | 0.010952708 | 0.014637493 |
| GBChow7 | 0.100251582 | 0.004726691 | 0.058524561 | 0.018906762 | 0.008945135 | 0 | 0.035526416 | 0.000432009 | 0.010139514 | 0.010419049 |
| Chow1 | 0.000355772 | 0.079870905 | 0.104673325 | 0.114253767 | 0.002007573 | 0.00383726 | 0.019237122 | 0 | 0.019516658 | 0.027165765 |
| Chow2 | 0.000508246 | 0.103504358 | 0.073721125 | 0.087774135 | 0.001905924 | 0.004929989 | 0.009478793 | 0 | 0.028665091 | 0.012096262 |
| Chow3 | 0.001296028 | 0.085309141 | 0.019643719 | 0.043302584 | 0.001855099 | 0.006378491 | 0.006708851 | 2.54E-05 | 0.013621001 | 0.010851058 |
| Chow4 | 0.000609896 | 0.161291962 | 0.088206145 | 0.081116109 | 0.001880511 | 0.004015146 | 0.006683439 | 0 | 0.013468527 | 0.00889431 |
| Chow5 | 0.000482834 | 0.123707148 | 0.080887398 | 0.093339432 | 0.006098956 | 0.02843638 | 0.00932632 | 0 | 0.022566136 | 0.010444461 |
| Chow6 | 0.001016493 | 0.145612564 | 0.112805265 | 0.076872252 | 0.010596935 | 0.041295012 | 0.017356611 | 0 | 0.009377144 | 0.016899189 |
| Chow7 | 0.01471373 | 0.144875607 | 0.071484842 | 0.079693019 | 0.002337933 | 0.005463648 | 0.030875962 | 0 | 0.01888135 | 0.011028945 |
